# Pediatric Out-of-Hospital Cardiac Arrest in a Physician-Staffed EMS System: A 13-Year Retrospective Descriptive Study from Southern Italy

**DOI:** 10.3390/jcdd13040170

**Published:** 2026-04-16

**Authors:** Luca Gregorio Giaccari, Gaetano Tammaro, Nicola D’Angelo, Daniele Antonaci, Eva Epifani, Luciana Mascia, Maria Caterina Pace, Vincenzo Pota, Pasquale Sansone

**Affiliations:** 1 Service of Anesthesia and Intensive Care, Vito Fazzi Hospital, 73100 Lecce, Italy; gaetanotammaro75@gmail.com (G.T.); evaepifani25@gmail.com (E.E.); 2 Department of Woman, Child, General and Specialized Surgery, University of Campania “Luigi Vanvitelli”, 80138 Naples, Italy; mariacaterina.pace@unicampania.it (M.C.P.); vincenzo.pota@unicampania.it (V.P.); pasquale.sansone@unicampania.it (P.S.); 3 Emergency Medical Service 118, Local Health Authority (ASL) Lecce, 73100 Lecce, Italy; nicola.dargenio@tiscali.it; 4 Presidio Ospedaliero Veris Delli Ponti, Scorrano, ASL Lecce, 73020 Scorrano, Italy; danieleantonaci.17@gmail.com; 5 Department of Experimental Medicine, University of Salento, 73100 Lecce, Italy; luciana.mascia@unisalento.it

**Keywords:** pediatric cardiac arrest, out-of-hospital, EMS, ventricular fibrillation, ROSC

## Abstract

**Background:** Pediatric out-of-hospital cardiac arrest (OHCA) is rare and associated with poor outcomes. Evidence from physician-staffed EMS systems remains limited. This study aimed to describe the incidence, presenting rhythms, EMS response intervals, and outcomes of pediatric OHCA, and to describe incidence, presenting rhythms, EMS response intervals, and prehospital outcomes in a local physician-staffed EMS system. **Methods:** We conducted a retrospective study of all pediatric (0–17 years) OHCA cases managed by the ASL Lecce physician-staffed EMS (southern Italy) between 2013 and 2025. Data were abstracted from standardized records. Variables included demographics, initial rhythm, EMS response intervals, temporal patterns, and return of spontaneous circulation (ROSC). The primary outcome was ROSC during prehospital care. **Results:** Twenty-seven cases were identified, corresponding to a cumulative incidence of 22.9 per 100,000 children over the study period (annualized incidence 1.73 per 100,000 children-year). Mean age was 11.9 ± 5.5 years (median 15); 59% were male. Initial rhythms were asystole in 81% and ventricular fibrillation (VF) in 19%; no pulseless ventricular tachycardia (pVT) or pulseless electrical activity (PEA) were recorded. Five patients had shockable rhythms, with seven shocks delivered overall. Mean time intervals were: event-to-call 1.0 ± 0.6 min, call-to-arrival 10.3 ± 4.1 min, event-to-arrival 11.3 ± 4.4 min. Arrests clustered during daytime (63%) and summer (41%). ROSC occurred in three patients (11%), two with VF and one with asystole; all arrests with ROSC were daytime events. In descriptive comparisons, ROSC cases showed a shorter call-to-arrival interval (T1–T2), whereas no consistent pattern was observed across all prehospital time intervals. **Conclusions:** Pediatric OHCA in this Italian physician-staffed EMS was infrequent, usually presented with asystole, and rarely achieved ROSC. Shockable rhythms were associated with better outcomes. Given the small sample size, findings related to response times should be interpreted with caution. System preparedness should include pediatric-specific training, early defibrillation access, and multicenter registries to improve care and track outcomes.

## 1. Introduction

Out-of-hospital cardiac arrest (OHCA) in children is a rare but devastating event associated with extremely poor outcomes. Despite advances in resuscitation science and prehospital care systems, survival after pediatric OHCA remains substantially lower than in adults. The incidence of pediatric OHCA has been estimated at 8–20 per 100,000 person-years, yet survival to hospital discharge seldom exceeds 10%, and favorable neurological outcomes are even less frequent [1,2].

Unlike adults, in whom ventricular fibrillation and acute coronary syndromes represent the predominant causes of OHCA, children more often present with asystole or pulseless electrical activity (PEA), typically secondary to respiratory failure, trauma, or sudden unexplained etiologies [3,4]. This fundamental difference in pathophysiology carries critical implications for resuscitation strategies, particularly regarding the role and timing of defibrillation and the likelihood of achieving return of spontaneous circulation (ROSC).

Several factors have been shown to influence survival after pediatric OHCA, including age, location of the arrest, presence of bystander cardiopulmonary resuscitation (CPR), initial rhythm, and emergency medical services (EMS) response intervals [5,6,7]. However, despite the availability of registry-based studies, the evidence remains heterogeneous, often reflecting regional differences in EMS organization, population characteristics, and resuscitation practices.

Most available evidence on pediatric OHCA derives from North American and Asian registries, with limited contributions from southern Europe. Furthermore, the majority of published data concern paramedic-based EMS, while systems with routine physician presence are less frequently reported [1,2,3,4,5,6,7]. This distinction is relevant because physician-staffed EMS models—such as those operating in Italy—may differ from paramedic-based systems in available interventions, response capabilities, and potentially outcomes.

In Italy, the EMS (Servizio di Emergenza Urgenza 118) is physician-staffed, yet regional epidemiology of pediatric OHCA remains scarcely documented. No prior studies have comprehensively described incidence, patient characteristics, and prehospital outcomes in this context.

Despite the existence of international registries, data from Mediterranean physician-staffed EMS systems remain extremely limited, and regional epidemiological patterns may differ due to organizational and demographic factors. To address this knowledge gap, we conducted a 13-year retrospective analysis of pediatric OHCA cases managed by the physician-staffed EMS system of ASL Lecce in southern Italy. The aim of this study was to provide a descriptive overview of pediatric OHCA cases managed in this regional EMS system, including incidence estimates, presenting rhythms, operational timings, and prehospital outcomes.

## 2. Materials and Methods

### 2.1. Study Design and Setting

We conducted a retrospective observational study of all pediatric OHCA cases attended by the Servizio di Emergenza Urgenza 118 within the Azienda Sanitaria Locale (ASL) di Lecce, Apulia region, southern Italy, between 1 January 2013 and 16 March 2025. The retrospective observational study complied with the principles of the Declaration of Helsinki, and ethical approval was obtained from the local committee of the “Vito Fazzi” Hospital (protocol no. 14/2018, approval date 15 January 2018). According to the institutional policies, no additional IRB approval was required for the extension of the retrospective data collection period until 16 March 2025, as the study design remained unchanged and the approval covered ongoing retrospective data analysis of eligible patients within the institution.

### 2.2. Inclusion and Exclusion Criteria

All patients aged 0–17 years who experienced an OHCA and for whom EMS activation occurred were eligible. Patients with in-hospital cardiac arrest or those without attempted resuscitation were excluded. Only the first rhythm documented by EMS personnel upon arrival at the scene was used for rhythm classification.

### 2.3. Data Collection

Data were abstracted from EMS records and standardized cardiac arrest report forms. Data collection and reporting followed the Utstein-style recommendations for reporting out-of-hospital cardiac arrest. Variables collected included demographic data (age, sex), event characteristics (location, time of day, season), prehospital time intervals (from event onset to EMS call [T0–T1], from call to EMS arrival [T1–T2], and total event-to-arrival interval [T0–T2]), presenting rhythm, number of shocks delivered, and resuscitation outcomes. Information regarding bystander cardiopulmonary resuscitation (CPR), witnessed status of the arrest, use of automated external defibrillators (AEDs), and underlying etiology was incompletely documented in the EMS registry and therefore could not be reliably analyzed. The time of event onset (T0) was derived from EMS documentation. In unwitnessed events, the estimation of T0 was based on retrospective witness or bystander reports and is therefore subject to recall bias, rounding, and documentation variability.

Rhythm classification followed European Resuscitation Council (ERC) guidelines [8]. Initial rhythms were classified into four categories: ventricular fibrillation (VF), pulse-less ventricular tachycardia (pVT), pulseless electrical activity (PEA), and asystole (AS), according to ERC definitions. Rhythm classification was based exclusively on the first rhythm documented by EMS upon arrival at the scene. ROSC was defined as any restoration of spontaneous circulation documented by EMS personnel. Data on duration or sustainability of ROSC were not consistently available and therefore could not be analyzed.

For temporal analyses, the 24 h day was divided into daytime (08:00–19:59) and nighttime (20:00–07:59) periods. In addition to this dichotomous comparison, circadian variation was explored by analyzing the distribution of initial rhythms across individual hours of the day (00:00–23:59). Events were also categorized according to weekday versus weekend occurrence. Seasonal variation was assessed by grouping cases into four meteorological seasons: spring (March–May), summer (June–August), autumn (September–November), and winter (December–February).

### 2.4. Outcomes

The primary outcome was any documented ROSC during prehospital EMS care. Longer-term outcomes, including survival to hospital discharge, 30-day survival, and neurological status, were not available from the EMS dataset. Secondary outcomes included distribution of rhythms, temporal patterns of OHCA, and associations between ROSC and operational timings.

### 2.5. Statistical Analysis

Continuous variables are presented as mean ± standard deviation (SD) or median (interquartile range), as appropriate. Categorical variables are reported as counts and percentages.

Because of the limited number of cases and outcome events, the statistical approach was intentionally descriptive. Any subgroup analyses were exploratory in nature and are reported solely to generate hypotheses rather than to support inferential conclusions. Comparisons between groups (ROSC vs. non-ROSC and daytime vs. nighttime events) were explored using the Mann–Whitney U test for continuous variables and Fisher’s exact test for categorical variables when appropriate.

Temporal patterns (hour of day and seasonal distribution) were analyzed descriptively to explore potential clustering of events.

Due to the limited sample size, statistical analyses should be interpreted as exploratory and hypothesis-generating rather than confirmatory. Given the extremely small number of ROSC events, inferential statistical comparisons should therefore be interpreted with caution.

All analyses were performed using R version 4.5.2 (R Foundation for Statistical Computing, Vienna, Austria) and IBM SPSS Statistics version 27 (IBM Corp., Armonk, NY, USA).

The cumulative incidence of pediatric OHCA over the study period was calculated using the resident pediatric population (0–17 years) of the province of Lecce as the denominator. According to data from the Italian National Institute of Statistics (ISTAT), the pediatric population was 115,882 residents in 2023. As yearly population data for the entire study period were not consistently available, the 2023 population was used as a proxy denominator. Therefore, incidence estimates should be interpreted as approximate and not as precise person-time–based rates. Accordingly, the resulting estimate should be considered a crude approximation of local frequency rather than a true person-time–based incidence rate.

## 3. Results

We observed 27 pediatric OHCA cases during the study period, corresponding to an approximate cumulative incidence of 22.9 per 100,000 children and an estimated annualized incidence of 1.73 per 100,000 children-year. Baseline characteristics of the study population are summarized in Table 1.

The mean age of patients was 11.9 ± 5.5 years, with a median of 15 years (range 0–17). 16 patients (59.3%) were males and 11 (40.7%) females. AS was observed in 22 cases (81.5%), and VF in 5 cases (18.5%). No cases of pVT or PEA were recorded. A total of 7 shocks were delivered among patients with shockable rhythms (*n* = 5), with a mean of 1.4 ± 0.9 shocks per patient (range 1–3).

Across the entire cohort, the mean intervals were approximately 1.0 ± 0.6 min for event onset to call activation (T0–T1), 10.3 ± 4.1 min for dispatch-to-arrival (T1–T2), and 11.3 ± 4.4 min for the overall event-to-arrival interval (T0–T2). These values should be interpreted as descriptive estimates, particularly for T0-based intervals, given the inherent uncertainty in the determination of event onset time.

**Figure 1:**
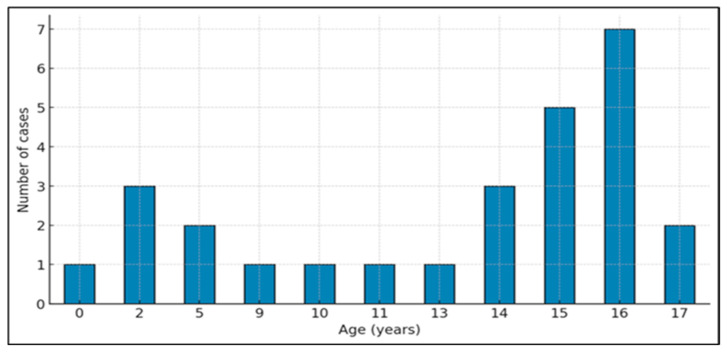
Age distribution of pediatric OHCA.

Age: As shown below (Figure 1), the age distribution was heterogeneous across the pediatric age spectrum. The highest incidence was observed at 16 years (7 cases, 25.9%), followed by 15 years (5 cases, 18.5%), 2 years (3 cases, 11.1%), and 14 years (3 cases, 11.1%). Two cases were reported at 5 and 17 years (7.4% each), while single cases (3.7%) occurred at 0, 9, 10, 11, and 13 years.

**Figure 2:**
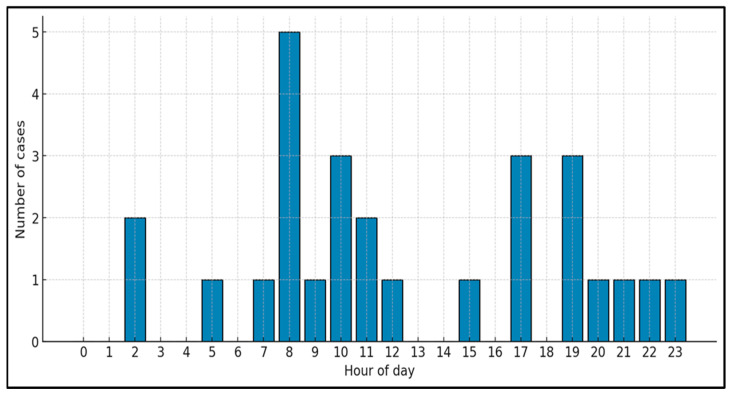
Hourly distribution of pediatric OHCA.

Time of day: Seventeen cases (63.0%) occurred during the daytime period (08:00–20:00) and 10 (37.0%) during nighttime (20:00–08:00). Hourly analysis suggested a non-uniform distribution across the 24 h cycle, with apparent peaks in the morning and late afternoon. The highest number of events occurred at 08:00 (5 cases, 18.5%), followed by 10:00, 17:00, and 19:00 (3 cases each, 11.1%). The remaining hours accounted for one or no events (Figure 2).

Seasons: Events were not evenly distributed across seasons. Events appeared to occur more frequently during summer (11 cases, 40.7%), followed by winter (8 cases, 29.6%), autumn (6 cases, 22.2%), and spring (2 cases, 7.4%), suggesting a potential seasonal pattern.

Dispatch and arrival times: Comparison of response times between daytime and nighttime events showed no significant differences. Analysis was performed on cases with complete time-interval data (daytime *n* = 17; nighttime *n* = 10). The mean T0–T1 interval was 1.0 ± 0.67 min during the day and 1.0 ± 0.53 min at night (Mann–Whitney U = 73.0, *p* = 0.86). The mean T1–T2 interval was 10.3 ± 4.0 min by day and 10.5 ± 4.4 min at night (U = 74.0, *p* = 0.94). The overall T0–T2 interval was 11.4 ± 4.2 min during the day and 11.2 ± 4.7 min at night (U = 73.0, *p* = 0.89).

ROSC: As shown in Table 2, three patients (11.1%) achieved ROSC. Their mean age was 9.0 ± 7.8 years (range 0–16), and all were male. ROSC occurred in two patients presenting with ventricular fibrillation and one with asystole. All ROSC events occurred during the daytime period. To provide greater clinical context, all three ROSC cases occurred during daytime EMS missions and represented the only patients in the cohort with documented prehospital restoration of spontaneous circulation. The T0–T1 interval was similar between groups, with a mean of approximately 1.0 ± 0.7 min in the non-ROSC group (*n* = 24) and 1.0 ± 0.0 min in the ROSC group (*n* = 3; *t*-test *p* = 0.74; Mann–Whitney *p* = 0.97). Given the small sample size and the uncertainty in T0 estimation, these values should be interpreted cautiously and considered descriptive only. The T1–T2 interval averaged 10.54 ± 3.12 min in non-ROSC patients versus 8.67 ± 1.15 min in ROSC patients (*t*-test *p* = 0.29; Mann–Whitney *p* = 0.25). In descriptive comparisons, the T0–T1 interval was similar between groups. The T1–T2 interval was numerically shorter in ROSC patients (8.67 ± 1.15 min) than in non-ROSC patients (10.54 ± 3.12 min). However, the total T0–T2 interval was numerically longer in ROSC cases (12.33 ± 1.53 min) than in non-ROSC cases (11.21 ± 4.42 min) (*t*-test *p* = 0.27; Mann–Whitney *p* = 0.21). Given the very small number of ROSC events, these findings should be interpreted as descriptive only. In the ROSC group, the time from event onset to ROSC (T0–ROSC) was 26.0 ± 7.94 min (range 17–32).

## 4. Discussion

This study provides a detailed description of pediatric OHCA within a physician-staffed EMS system in southern Italy over a 13-year period. Our findings confirm the rarity of pediatric OHCA and the persistently poor outcomes associated with this condition.

When related to the underlying pediatric population, the 27 OHCA cases observed in our region correspond to an approximate cumulative frequency corresponding to 22.9 cases per 100,000 children over the study period, with an estimated annualized value of 1.73 per 100,000 children, interpreted as a crude approximation. This estimate is substantially lower than incidence rates reported in most international registries, which typically range from 8 to 20 per 100,000 children-year [1,2]. However, this comparison should be interpreted with caution. Our incidence calculation was based on a single-year population denominator, used as a proxy for the entire 13-year study period, and therefore does not represent a true person-time–based incidence. Differences in case ascertainment, population structure, and EMS system organization may partially explain this discrepancy. For these reasons, the incidence reported in this study should be considered an approximate local estimate, and direct comparisons with registry-based studies should be interpreted carefully. Given the use of a proxy denominator, this estimate should not be interpreted as directly comparable to registry-based incidence rates derived from person-time data.

Our findings are broadly consistent with data from large international registries. Data from the European Registry of Cardiac Arrest (EuReCa) confirm the rarity of pediatric OHCA and the predominance of non-shockable rhythms in this population [9]. Similarly, registry analyses from European EMS systems and large international datasets such as Get With The Guidelines–Resuscitation (GWTG-R) consistently demonstrate low survival rates and emphasize the importance of early recognition, bystander CPR, and rapid EMS response in improving outcomes [6,10]. These observations support the external validity of our results while highlighting the need for regional epidemiological data, particularly from Mediterranean EMS systems where published evidence remains limited.

Several key observations emerge from this study. First, a key finding of this study is the overwhelming predominance of non-shockable rhythms. Asystole accounted for more than 80% of cases, whereas ventricular fibrillation (VF) was present in fewer than one fifth of patients. The absence of PEA and pulseless VT in this series should be interpreted cautiously. Because rhythm classification was based on the first rhythm documented by EMS on arrival, some patients may have evolved to asystole before EMS assessment. This pattern differs markedly from adult OHCA, where shockable rhythms are more frequent and are commonly associated with primary cardiac etiologies. In children, cardiac arrest is more often secondary to respiratory failure, trauma, or other non-cardiac conditions, which likely explains the predominance of asystole observed in our cohort [3,6,11]. Importantly, although shockable rhythms were uncommon, the majority of ROSC cases occurred in patients presenting with VF, reinforcing the well-established prognostic value of shockable rhythms even in pediatric populations [1].

Second, the overall ROSC rate of 11.1% is in line with international data but remains alarmingly low [1,6]. Although survival to hospital discharge and neurological outcomes were not available, ROSC remains a key intermediate outcome and reflects both patient factors and system-level determinants, including early recognition of cardiac arrest, bystander intervention, and EMS response times [12].

Third, another notable observation is the temporal distribution of events. Pediatric OHCA occurred more frequently during daytime hours and appeared to cluster during the summer months. While the small sample size precludes firm conclusions, these patterns may reflect differences in daily activity levels, environmental exposures, or social behaviors. The predominance of cases among adolescents further supports the hypothesis that older children may have distinct risk exposures, including sports-related activities or previously undiagnosed cardiac conditions such as inherited channelopathies or cardiomyopathies [5,7,13]. However, given the limited number of events, these temporal patterns may reflect random variation and should be interpreted descriptively only.

Fourth, although prehospital timing variables were explored, the observed pattern was not consistent across all response intervals. While the call-to-arrival interval (T1–T2) was numerically shorter in ROSC cases, the total event-to-arrival interval (T0–T2) was numerically longer. Given the extremely small number of ROSC events, no robust inference can be drawn regarding the association between EMS response intervals and outcomes. These observations should therefore be interpreted as descriptive and hypothesis-generating only. Interpretation of prehospital time intervals involving T0 should be cautious, as the estimation of event onset is inherently imprecise in retrospective datasets, particularly for unwitnessed arrests. Therefore, these variables should be considered approximate and primarily descriptive.

These findings also raise important considerations regarding system preparedness. Pediatric OHCA represents a very small fraction of EMS workload, yet it requires highly specialized skills, including pediatric airway management, vascular access, and age-specific drug dosing. Maintaining proficiency in these low-frequency, high-stakes scenarios is inherently challenging. Simulation-based training, standardized pediatric resuscitation protocols, and periodic team-based training may therefore play a crucial role in improving preparedness and performance during pediatric resuscitation events [14,15].

Limitations. This study has several limitations. First, the small sample size limits the statistical power of the analysis and restricts the ability to identify significant associations between clinical or operational variables and outcomes. As a result, the findings should be interpreted primarily as descriptive and hypothesis-generating. Second, the retrospective design introduces the potential for missing or incomplete data and limits control over confounding variables. In addition, several key Utstein variables—such as bystander cardiopulmonary resuscitation (CPR), witnessed arrest status, use of automated external defibrillators (AEDs), and underlying etiology—were not consistently recorded in the EMS database and therefore could not be included in the analysis. The absence of several core Utstein variables limits interpretation of the observed ROSC rate, restricts comparison with registry-based studies, and prevents meaningful analysis of determinants of outcome. Future prospective registries should systematically collect these data. Third, an additional limitation concerns the estimation of event onset time (T0). In cases that were not directly witnessed, T0 was reconstructed from bystander or witness reports and EMS documentation, which may be affected by recall bias, rounding, and reporting inaccuracies. As a result, time intervals derived from T0 (including T0–T1, T0–T2, and T0–ROSC) should be interpreted with caution, as their apparent numerical precision may not reflect the underlying data accuracy. Fourth, outcomes were limited to ROSC during prehospital care, and information on survival to hospital discharge or neurological outcomes was not available. Because ROSC is an intermediate prehospital outcome, the absence of longer-term survival and neurological data substantially limits the clinical interpretability of the findings. Finally, as a single-system study conducted within one regional EMS network, the findings may reflect local EMS organizational characteristics and may not be fully generalizable to other settings.

Despite these limitations, our findings add to the growing body of evidence that pediatric OHCA requires urgent focus. Early recognition, rapid EMS activation, and immediate initiation of CPR are critical. Expanding public awareness, increasing bystander CPR training, and ensuring availability of AEDs in schools, sports facilities, and public places could improve outcomes. Multicenter, prospective registries are necessary to validate prognostic factors, assess neurological outcomes, and inform tailored guidelines for pediatric resuscitation.

## 5. Conclusions

Pediatric out-of-hospital cardiac arrest remains a rare but devastating event with poor outcomes. In this physician-staffed EMS system, most arrests presented with non-shockable rhythms and ROSC was infrequent. The findings are primarily descriptive and should be interpreted in light of the lack of longer-term survival and neurological outcomes. However, they highlight the importance of system preparedness, including pediatric-specific training, rapid EMS response, and improved community access to early CPR and defibrillation. Large multicenter registries are needed to better characterize the epidemiology of pediatric OHCA and to identify modifiable factors that may improve survival and neurological outcomes.

## Figures and Tables

**Table 1:** Patient and event characteristics of pediatric OHCA cases.

	Total (*n* = 27)
**Age, mean ± SD (years)**	11.9 ± 5.5
**Age, median (range)**	15 (0–17)
**Male sex, *n* (%)**	16 (59.3%)
**Female sex, *n* (%)**	11 (40.7%)
**Initial rhythm**	
— AS	22 (81.5%)
— VF	5 (18.5%)
— pVT	0
— PEA	0
**Shock delivered, *n* (%)**	5 (18.5%)
**Number of shocks (mean ± SD)**	1.4 ± 0.9
**Time intervals (minutes)**	
— Event-to-call (T0–T1)	1.0 ± 0.62
— Call-to-arrival (T1–T2)	10.33 ± 4.12
— Event-to-arrival (T0–T2)	11.33 ± 4.38
**Time of day**	
— Daytime	17 (63.0%)
— Nighttime	10 (37.0%)
**Season**	
— Spring	2 (7.4%)
— Summer	11 (40.7%)
— Autumn	6 (22.2%)
— Winter	8 (29.6%)
**ROSC achieved, *n* (%)**	3 (11.1%)

**Table 2:** Characteristics of ROSC cases.

Case	Sex	Age (Years)	Initial Rhythm	T0–T1 (min)	T1–T2 (min)	T0–T2 (min)	T0–ROSC (min)	Shocks
1	M	0	AS	~1.0	~13	~14	~32	-
2	M	14	VF	~1.0	~ 8	~9	~17	1
3	M	13	VF	~1.0	~ 5	~6	~29	3

## Data Availability

The datasets generated and analyzed during the current study are available from the corresponding author on reasonable request.

## Abbreviations

AED	Automated External Defibrillator
AS	Asystole
ASL	Azienda Sanitaria Locale
CPR	Cardiopulmonary Resuscitation
EMS	Emergency Medical Services
ERC	European Resuscitation Council
EuReCa	European Registry of Cardiac Arrest
GWTG-R	Get With The Guidelines–Resuscitation
IRB	Institutional Review Board
ISTAT	Italian National Institute of Statistics
OHCA	Out-of-Hospital Cardiac Arrest
PEA	Pulseless Electrical Activity
pVT	Pulseless Ventricular Tachycardia
ROSC	Return of Spontaneous Circulation
SD	Standard Deviation
T0–T1	Event-to-call interval
T0–T2	Event-to-arrival interval
T1–T2	Call-to-arrival interval
VF	Ventricular Fibrillation
